# ULK2 Is a Key Pro-Autophagy Protein That Contributes to the High Chemoresistance and Disease Relapse in FLT3-Mutated Acute Myeloid Leukemia

**DOI:** 10.3390/ijms25010646

**Published:** 2024-01-04

**Authors:** Justine Lai, Claire Yang, Chuquan Shang, Will Chen, Michael P. Chu, Joseph Brandwein, Raymond Lai, Peng Wang

**Affiliations:** 1 Department of Medicine, Division of Hematology, University of Alberta, Edmonton, AB T6G 2R3, Canada; jlai@ualberta.ca (J.L.); mpchu@ualberta.ca (M.P.C.); jbrandwe@ualberta.ca (J.B.); 2 Department of Laboratory Medicine and Pathology, University of Alberta, Edmonton, AB T6G 2R3, Canada; yiling18@ualberta.ca (C.Y.); chuquan@ualberta.ca (C.S.); will.chen@ualberta.ca (W.C.); 3 Department of Medical Oncology, Cross Cancer Institute, Edmonton, AB T6G 1Z2, Canada

**Keywords:** acute myeloid leukemia, cancer stemness, relapse, autophagy

## Abstract

We recently demonstrated that a small subset of cells in FLT3-mutated acute myeloid leukemia (AML) cell lines exhibit SORE6 reporter activity and cancer stem-like features including chemoresistance. To study why SORE6^+^ cells are more chemoresistant than SORE6^−^ cells, we hypothesized that these cells carry higher autophagy, a mechanism linked to chemoresistance. We found that cytarabine (Ara-C) induced a substantially higher protein level of LC3B-II in SORE6^+^ compared to SORE6^−^ cells. Similar observations were made using a fluorescence signal-based autophagy assay. Furthermore, chloroquine (an autophagy inhibitor) sensitized SORE6^+^ but not SORE6^−^ cells to Ara-C. To decipher the molecular mechanisms underlying the high autophagic flux in SORE6^+^ cells, we employed an autophagy oligonucleotide array comparing gene expression between SORE6^+^ and SORE6^−^ cells before and after Ara-C treatment. *ULK2* was the most differentially expressed gene between the two cell subsets. To demonstrate the role of ULK2 in conferring higher chemoresistance in SORE6^+^ cells, we treated the two cell subsets with a ULK1/2 inhibitor, MRT68921. MRT68921 significantly sensitized SORE6^+^ but not SORE6^−^ cells to Ara-C. Using our in vitro model for AML relapse, we found that regenerated AML cells contained higher ULK2 expression compared to pretreated cells. Importantly, inhibition of ULK2 using MRT68921 prevented in vitro AML relapse. Lastly, using pretreatment and relapsed AML patient bone marrow samples, we found that ULK2 expression was higher in relapsed AML. To conclude, our results supported the importance of autophagy in the relapse of FLT3-mutated AML and highlighted ULK2 in this context.

## 1. Introduction

Acute myeloid leukemia (AML) is an aggressive hematologic malignancy carrying a 5-year overall survival rate of only 30% [1]. Chemoresistance, which contributes to the development of refractory and relapsed diseases, is a significant challenge in the treatment of AML patients. Refractory disease, typically defined by a failure to induce complete remission, occurs in approximately 30% of patients who are treated with a curative intent [2]. Relapsed disease, typically defined by disease recurrence after a period of complete remission, occurs in approximately 50% of patients treated with a curative intent [3]. The mechanisms underlying AML relapse remain elusive, partly due to a lack of appropriate in vitro study models. Nonetheless, one of the hypotheses is that a very small proportion of treatment-resistant cells survive the initial chemotherapy, and after a period of remission, these cells provide the seeds for the return of full-blown disease. Accumulating evidence suggests that this small cell subset is enriched with cancer stem cells or cancer stem-like (CSL) cells. In this regard, a few prior studies have shown that CSL cells have significantly higher drug resistance compared to bulk cancer cells [4,5]. While it has been published that CSL cells in AML can be detected and/or purified by using various cell surface or cytoplasmic markers (e.g., CD34^+^/CD38^−^, CD200) and ALDH [6,7,8], we chose to study CSL cells in AML using the SORE6 (Sox2/Oct4 response element) reporter system, which contains six repeats of the consensus sequences for Sox2/Oct4 [5]. Unlike the abovementioned cell surface or cytoplasmic markers, SORE6 has a direct biological link to stemness, as it was originally designed to detect/quantify the activity of embryonic stem cells proteins known to play a central role in maintaining stemness and pluripotency [9]. The utility of the SORE6 reporter as an experimental tool to study CSL cells has also been tested and validated in various solid and hematologic cancers [10,11,12]. Using SORE6, we previously detected/purified a small subset of SORE6^+^ cells in two FLT3-mutated AML cell lines and found that SORE6^+^ cells have significantly higher CSL phenotype than the bulk SORE6^−^ cells, including chemoresistance [13]. In the same study, leveraging this SORE6^−^/SORE6^+^ dichotomy, we developed an in vitro AML relapse model, in which regeneration of AML cells was observed after a time period during which no viable cells could be identified using trypan blue; importantly, the regenerated cells were found to be enriched in SORE6^+^ cells, a finding that is supportive of the role of CSL cells in AML relapse [13].

The mechanisms underlying the high chemoresistance in CSL cells in AML are incompletely understood, although several possibilities have been identified including the upregulation of transporters (e.g., MDR1) to export cytotoxic drugs [14] and that of anti-apoptotic proteins such as Bcl-2 [15]. More recently, much attention has been given to the postulation of heightened macro-autophagy (hereafter referred to as “autophagy”) in CSL cells as a key mechanism of chemoresistance [16]. Autophagy, a process whereby intracellular components are degraded and recycled, has been shown to be cytoprotective in cancer cells [17]. There is also accumulating evidence that autophagy is closely linked to cancer stemness. Specifically, a study using ovarian cancer cells showed that inhibition of autophagy using chloroquine or ATG5 knockout effectively decreased the CSL features, including spheroid formation, tumorigenicity, and the expression of stem cell markers Sox2, Nanog, and Oct4, in a small cell subset characterized by the CD188^+^CD44^+^ immunophenotype [18]. AML stem cells, defined as CD34^+^/ROS^low^, have been shown to have elevated autophagy compared to CD34^+^/ROS^high^ cells, and inhibition of autophagy in AML stem cells resulted in decreased engraftment in mice [19]. In another AML study, leukemic stem cells, defined as CD34^+^CD38^−^, were shown to have heightened autophagy and increased chemoresistance upon treatment with JQ1, an epigenetic agent proposed to treat AML [16]. In view of the new evidence supporting a link between autophagy and AML cancer stem cells, we asked if autophagy contributes to the high chemoresistance in SORE6^+^ cells and the ‘in vitro relapse’ in our AML study model. If so, it might be possible to utilize our in vitro model to decipher the molecular events underlying the heightened autophagic response in the SORE6^+^ CSL cells.

In this study, we aimed to decipher the role of autophagy in the chemoresistance of CSL cells and ‘in vitro relapse’, and to use our study model to explore the mechanisms underlying the autophagy-mediated chemoresistance and ‘in vitro relapse’ of SORE6^+^ cells. We first determined that there is a significantly higher chemotherapy-induced autophagic response in SORE6^+^ cells, as compared to SORE6^−^ cells. Using chloroquine, an autophagic inhibitor, we found evidence that the heightened autophagic response in SORE6^+^ cells directly contributes to their higher chemoresistance and their relatively efficient regeneration in our in vitro model. Using an autophagy pathway-specific array, we then identified ULK2 as a key molecule underlying the enhanced autophagy response in SORE6^+^ cells.

## 2. Results

### 2.1. SORE6^+^ Cells Exhibit Higher Autophagic Flux than SORE6^−^ Cells

**Figure 1:**
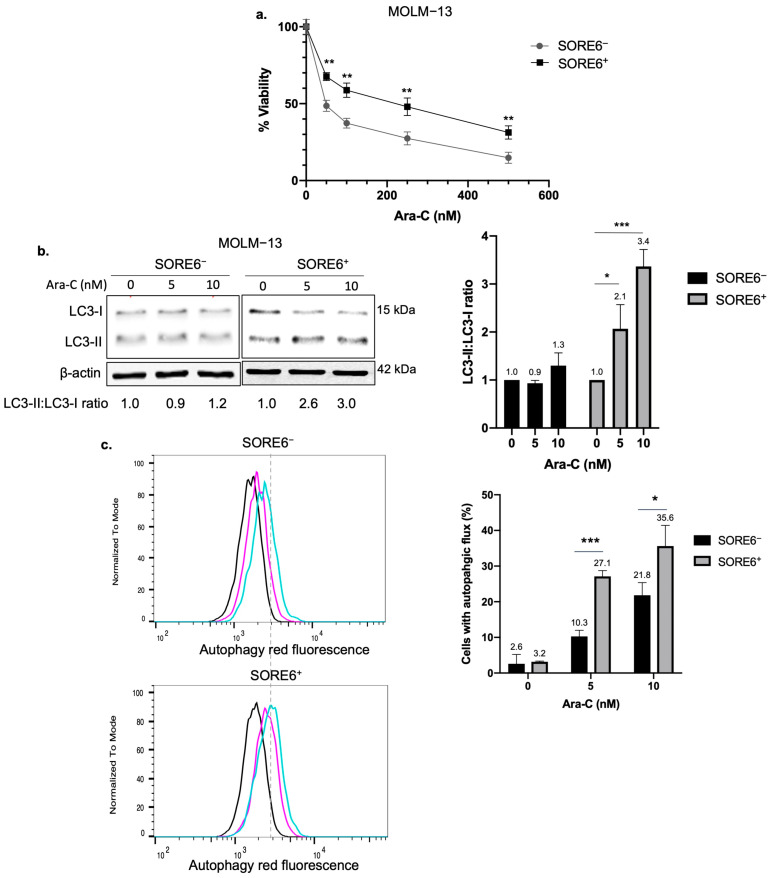
SORE6^+^ cells exhibit higher autophagic flux in response to Ara-C. (**a**) Cell viability of MOLM−13 SORE6^−^ and SORE6^+^ cells after treatment with increasing doses of Ara-C for 24 h. Cell viability was assessed using trypan blue and was performed in triplicates. (**b**) Western blots of LC3 in MOLM−13 SORE6^−^ and SORE6^+^ cells after treatment with 0, 5, or 10 nM of Ara-C, with 10 µM of chloroquine for 24 h. LC3-II and LC3-I bands were quantified with densitometry analysis using ImageJ. Data from three independent experiments are shown in the bar graph. (**c**) Flow cytometric analysis of MOLM−13 SORE6^−^ and SORE6^+^ cells subjected to the Autophagy Assay Red Detection Kit after treatment with 0, 5, or 10 nM of Ara-C for 24 h. Data shown as mean ± standard deviation. *p*-value calculated using a Student’s *t*-test, where * *p* < 0.05, ** *p* < 0.01, *** *p* < 0.001.

We recently found that purified SORE6^+^ cells derived from MOLM−13 and MV4−11, two AML cell lines carrying FLT3-ITD, displayed significantly higher CSL phenotype (such as resistance to Ara-C) compared to SORE6^−^ cells [13]. Using SORE6^−^/SORE6^+^ cells derived from MOLM−13, we found that the Ara-C inhibitory concentration at 50% (IC50) for SORE6^+^ cells was 172.9 nM, which is significantly higher than that of their SORE6^−^ counterparts (61.0 nM, *p* < 0.0001) (Figure 1a). Similar results were obtained when SORE6^+^/SORE6^−^ cells derived from MV4−11 were used (61.8 versus 29.0 nM, *p* < 0.001).

Since autophagy has been shown to contribute to chemoresistance in cancer cells, we asked whether the autophagic flux in SORE6^+^ cells is higher than that in SORE6^−^ cells. To assess the autophagy response, we employed Western blots to quantify the LC3-II:LC3:I protein ratio before and after Ara-C treatment. In the presence of chloroquine, SORE6^+^ cells derived from MOLM−13 showed substantially higher accumulation of LC3-II than SORE6^−^ cells. In a triplicate experiment, densitometric quantification of the LC3-II and LC3-I bands revealed that SORE6^+^ cells exhibited a mean 2.1-fold increase in the LC3-II:LC3-I ratio upon treatment of 5 nM of Ara-C for 24 h (*p* = 0.02), whereas the same treatment did not induce any significant change in SORE6^−^ cells (illustrated in Figure 1b). Similar results were obtained when 10 nM of Ara-C was used, with SORE6^+^ cells showing a mean 3.4-fold increase in the LC3-II:LC3-I ratio, while no significant change was observed in SORE6^−^ cells. To substantiate these observations, we also measured the autophagic response using a fluorescence signal-based assay. As shown in Figure 1c, treatment with Ara-C at 5 or 10 nM induced significantly higher autophagy in SORE6^+^ cells compared to SORE6^−^ cells. These experiments were then repeated by using SORE6^−^/SORE6^+^ cells derived from MV4−11, and similar results were obtained.

### 2.2. Inhibition of Autophagy Sensitizes SORE6^+^ Cells to Ara-C

**Figure 2:**
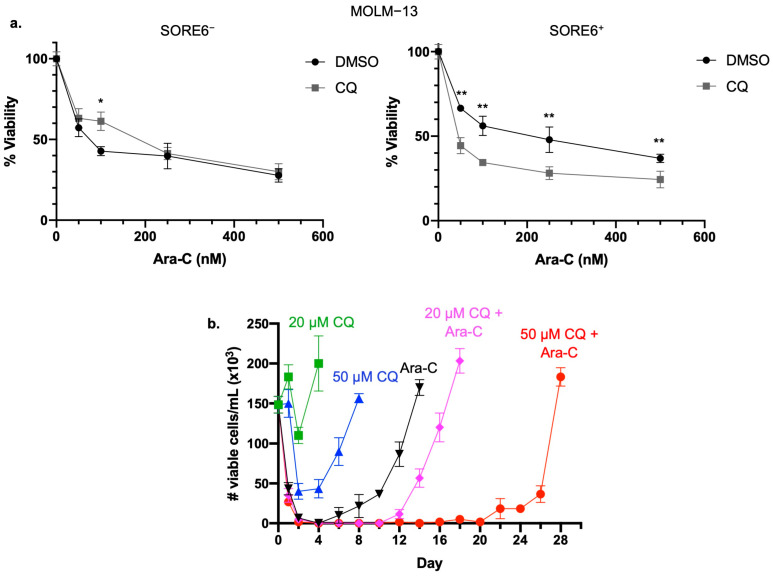
Inhibiting autophagy sensitizes SORE6^+^ cells to Ara-C treatment. (**a**) Cell viability of MOLM−13 SORE6^−^ and SORE6^+^ cells subjected to increasing doses of Ara-C, with or without 10 µM of chloroquine, for 24 h. Cell viability, assessed using trypan blue, was normalized to cells treated with DMSO, and three independent experiments were performed. Data reported as mean ± standard deviation. * denotes *p* < 0.05, ** *p* < 0.01, using Student’s *t* test. (**b**) Cell viability, assessed using trypan blue, in MV4−11 cells subjected to the in vitro relapse model treated with 500 nM Ara-C alone, 20 or 50 µM CQ alone, or the combination of Ara-C with 20 or 50 µM CQ.

We then asked if autophagy directly contributes to the higher chemoresistance in SORE6^+^ cells. As shown in Figure 2a, addition of chloroquine significantly decreased the IC50 from 179.3 to 57.5 nM (*p* = 0.009). In contrast, chloroquine treatment did not significantly change the IC50 in SORE6^−^ cells. Similar results were observed with SORE6^−^/SORE6^+^ cells derived from MV4−11, with the Ara-C IC50 in SORE6^+^ cells decreasing from 65.1 to 14.6 nM when chloroquine was added (*p* < 0.0001), while no significant decrease in the IC50 of SORE6^−^ cells was observed with the addition of chloroquine.

### 2.3. Inhibition of Autophagy Prolongs Time to In Vitro Relapse

We recently generated an in vitro model with features mimicking AML relapse [13]. Specifically, an ‘in vitro remission state’, defined by the absence of trypan blue-negative cells induced by the lowest required dose of Ara-C, is followed by regeneration of AML cells (i.e., ‘in vitro relapse’) after a period of time. In this model, the regenerated cells are highly enriched with SORE6^+^ cells, suggesting these CSL cells are the major contributors to the ‘in vitro relapse’. Given our findings that autophagy protects SORE6^+^ cells from Ara-C treatment, we asked if autophagy contributes to the ‘in vitro relapse’. As illustrated in Figure 2b, inhibition of autophagy using chloroquine significantly increased the duration of ‘in vitro remission’. Specifically, treatment with chloroquine in combination with Ara-C increased the time for cells to regenerate in a dose-dependent manner, i.e., time to reach the number of viable cells that were observed at the start of the experiment, from 14 days when treated with Ara-C alone to 18 days when treated with Ara-C combined with 20 µM chloroquine and 28 days when treated with Ara-C combined with 50 µM chloroquine.

### 2.4. ULK2 Is a Key Regulator of the Autophagic Flux in SORE6^+^ Cells

To decipher the molecular basis underlying the relatively high autophagy capacity of SORE6^+^ cells, we employed a commercially available oligonucleotide array that includes 84 autophagy-related genes. We first identified the differential gene expression in both SORE6^−^ and SORE6^+^ cells before and after they were treated with 10 nM of Ara-C, a level with which we observed a substantial difference in the autophagy response between these two cell subsets. We then compared the gene lists from the two cell subsets. We found that *ULK2* was the most differentially expressed gene between SORE6^+^ and SORE6^−^ cells after Ara-C treatment, with 13.4-fold higher expression in Ara-C treated SORE6^+^ compared to SORE6^−^ cells. The full list of analyzed genes and fold up- and downregulation between untreated and Ara-C-treated subsets is shown in Table S1.

**Figure 3:**
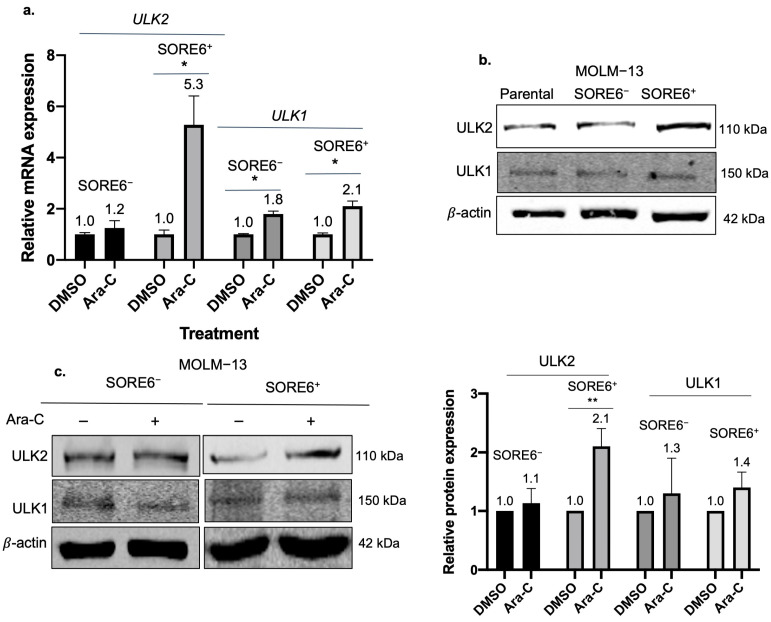
ULK2 is significantly upregulated after Ara-C treatment in SORE6^+^ cells. (**a**) Gene expression level of *ULK2* or *ULK1* in MOLM−13 SORE6^−^ and SORE6^+^ cells after Ara-C treatment for 24 h, normalized to cells treated with DMSO. Data reported as mean ± standard deviation (triplicate experiments). (**b**) Western blot analysis of ULK2 and ULK1 in MOLM−13 parental cells and SORE6^−^ and SORE6^+^ cell subsets at steady state. ULK2/*β*-actin and ULK1/*β*-actin ratios normalized to SORE6^−^ cell subsets. (**c**) Western blot analysis of ULK2 and ULK1 in MOLM−13 SORE6^−^ and SORE6^+^ subsets after treatment with 10 nM Ara-C for 24 h, as compared to DMSO treatment. Data from three independent experiments are shown in graph. * denotes *p* < 0.05, ** denotes *p* < 0.01 using Student’s *t* test.

In view of the known importance of ULK1, a homologue of ULK2, in the autophagy pathway [20], and our finding that *ULK2* is the most differentially expressed gene between the two cell subsets treated with Ara-C, we focused the remainder of this study on ULK2. Firstly, we validated the upregulation of *ULK2* induced by Ara-C using quantitative RT-PCR. Since ULK1 and ULK2 have been shown to have substantial structural similarities and functional redundancy [21], we included ULK1 for comparison. As shown in Figure 3a, after subjecting SORE6^−^/SORE6^+^ cells derived from MOLM−13 to 10 nM of Ara-C for 24 h, *ULK2* expression was found to be significantly higher in SORE6^+^ cells (mean = 5.3-fold, *p* = 0.02). In comparison, no significant change in *ULK2* expression was observed in SORE6^−^ cells. The expression of *ULK1* was also found to be significantly increased in SORE6^+^ cells, although the increment was relatively small (i.e., 2.1-fold) compared to that of ULK2 (i.e., 5.3-fold).

We then performed Western blot to analyze ULK1/2 protein expression in SORE6^−^/SORE6^+^ cells at steady state and after treatment with Ara-C. As shown in Figure 3b, ULK2 was substantially higher in SORE6^+^ cells at steady state, whereas no substantial differences were observed in ULK1. As illustrated in Figure 3c, we found that ULK2 was substantially upregulated in SORE6^+^ cells treated with 10 nM Ara-C. In contrast, ULK2 was not appreciably changed in SORE6^−^ cells treated with Ara-C. Changes in ULK1 induced by Ara-C in both cell subsets were relatively minimal. Taken together, these findings highlight the Ara-C-induced upregulation of ULK2, but not ULK1, suggesting that ULK2 might be a key contributor to the preferentially robust autophagy response in SORE6^+^ cells. Similar experiments were performed in MV4−11 cells, and similar findings were derived.

### 2.5. ULK2 Inhibition Sensitizes SORE6^+^ Cells to Ara-C Treatment

**Figure 4:**
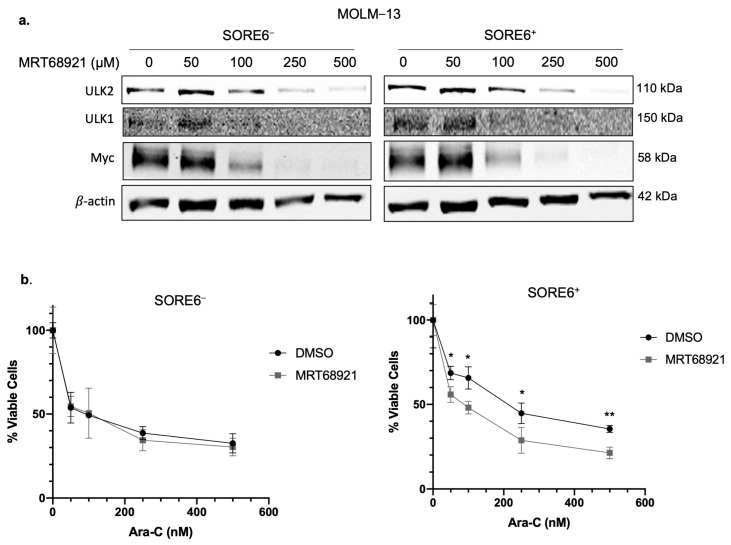
ULK1/2 inhibition sensitizes SORE6^+^ cells to Ara-C treatment. (**a**) Western blot analysis of ULK2, ULK1, and Myc in SORE6^−^ and SORE6^+^ cells derived from MOLM−13 after treatment with 50–500 nM MRT68921 for 24 h. (**b**) Cell viability of SORE6^−^ and SORE6^+^ subsets derived from MOLM−13 after treatment with 0, 50, 100, 250, or 500 nM Ara-C, either in the presence of 250 nM MRT68921 or DMSO, for 24 h. Cell viability was normalized to the DMSO treatment group. Data are reported as mean ± standard deviation (triplicate experiments). * *p* < 0.05, ** *p* < 0.01, using Student’s *t* test.

We further evaluated whether ULK2 plays a direct role in conferring the higher Ara-C resistance and autophagic flux in SORE6^+^ cells. ULK1/2 activity was inhibited by using the pharmacologic agent MRT68921. As shown in Figure 4a, the expression of ULK2 was dramatically decreased by MRT68921 in a dose-dependent manner. As shown in Figure 4b, pharmacological inhibition of ULK2 in SORE6^+^ cells derived from MOLM−13 led to a significant reduction in their IC50 to Ara-C (194.0 to 88.0 nM, *p* = 0.003). In contrast, the same treatment did not significantly change the IC50 in SORE6^−^ cells.

### 2.6. ULK2 and Myc Form a Positive Feedback Loop

**Figure 5:**
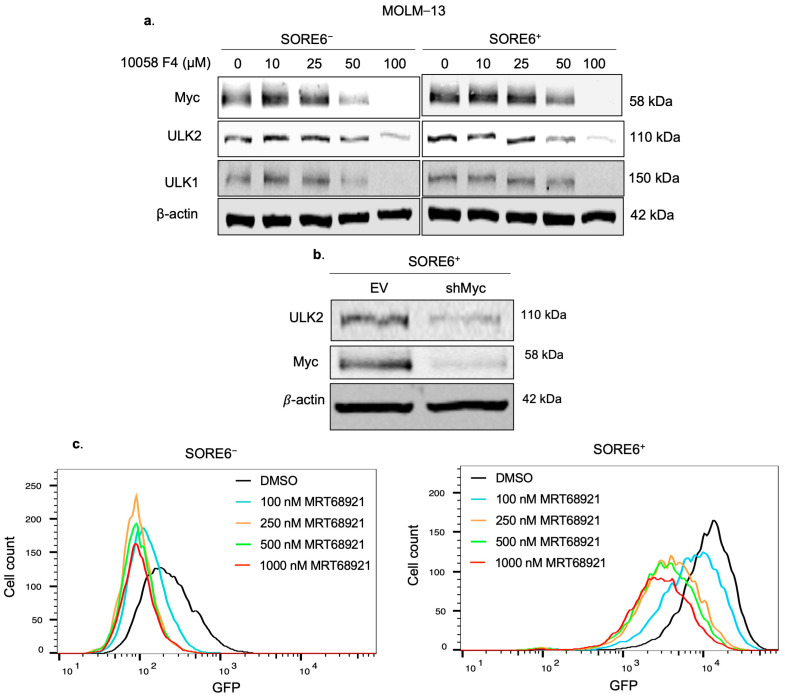
ULK2 and Myc form a positive feedback loop. (**a**) Western blot analysis of Myc, ULK2, and ULK1 in MOLM−13 SORE6^−^ and SORE6^+^ subsets after treatment with 10–100 µM of 10058-F4 for 24 h. (**b**) Western blot analysis of ULK2 and Myc in MOLM−13 SORE6^+^ cells transduced with shMyc or an empty vector (EV). (**c**) GFP levels, assessed using flow cytometry, of MOLM−13 SORE6^+^ cells treated with increasing doses of MRT68921 for 24 h.

Myc has been implicated as a regulator of cancer stemness, as it has been shown to promote pluripotency [9]. Additionally, we previously found that Myc is higher expressed in SORE6^+^ compared to SORE6^−^ cells, and that Myc can regulate SORE6 activity, as inhibition of Myc by shRNA or pharmacological inhibition decreased green fluorescent protein (GFP) expression in SORE6^+^ cells [13]. Given the evidence supporting Myc as a regulator of cancer stemness, and our findings that ULK2 may promote the CSL feature of chemoresistance in SORE6^+^ cells, we asked if the expression level of ULK2 is regulated by Myc. As shown in Figure 5a, pharmacological inhibition of Myc with 10058-F4 resulted in an appreciable decrease in the protein expression of ULK2. These results may explain the preferential high expression of ULK2 in SORE6^+^ cells, since the high Myc protein level in these cells may directly contribute to the upregulation of ULK2. Similarly, knockdown of Myc using shRNA showed a dramatic decrease in ULK2 expression in SORE6^+^ cells (Figure 5b).

As shown in Figure 4a, inhibition of ULK2 using MRT68921 substantially decreased Myc protein expression. In keeping with the concept that Myc is a key driver of the SORE6 activity in SORE6^+^ cells, inhibition of ULK2 activity using MRT68921 in SORE6^+^ cells showed a significant decrease in %SORE6^+^ cells in a dose-dependent manner, from 94.0 ± 4.2 to 78.5 ± 2.5% when treated with 100 nM, 55.2 ± 2.8% when treated with 250 nM, 46.6 ± 5.1% when treated with 500 nM, and 41.3 ± 1.8% when treated with 1000 nM (Figure 5c). Taken together, these results suggest that ULK2 and Myc form a positive feedback loop in FLT3-mutated AML cells.

### 2.7. ULK2 Is Critical for the Regeneration of Ara-C Treated Cells

**Figure 6:**
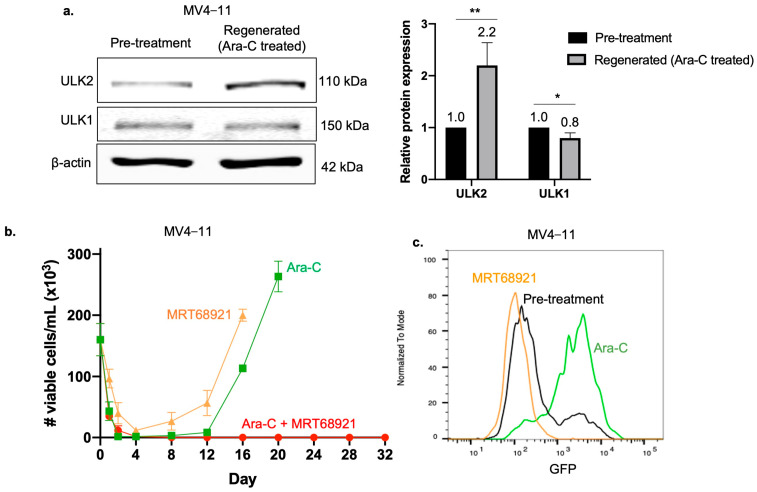
ULK2 contributes to cell regeneration from Ara-C treatment. (**a**) Western blots of ULK1 and ULK2 in MV4−11 cells at ‘in vitro relapse’ after Ara-C treatment compared to cells at pretreatment. Data from three independent experiments are shown in the bar graph. ULK2 and ULK1 expression were normalized to β-actin. * denotes *p* < 0.05, ** denotes *p* < 0.01. (**b**) Cell viability assessed using trypan blue in MV4−11 cells subjected to the in vitro relapse model treated with Ara-C alone, MRT68921 alone, or combination of Ara-C + MRT68921. Three independent experiments were performed. (**c**) Flow cytometry analysis of GFP levels in cells that regenerated from Ara-C treatment or MRT68921 treatment, compared to cells at pretreatment.

In light of our evidence that ULK2 is a key regulator of autophagic-induced chemoresistance of SORE6^+^ cells, we asked if ULK2 plays an important role in the ‘in vitro relapse’. Firstly, we examined the protein expression of ULK2 in MV4−11 regenerated cells. Cells pooled in a 9:1 ratio of SORE6^−^:SORE6^+^ subsets were subjected to Ara-C treatment. The cells regenerated (i.e., achieved the same number of viable cells present at the start of the experiment) 14 days after reaching ‘in vitro remission’. As shown in Figure 6a, there was a substantial increase in ULK2 protein level in regenerated cells compared to cells collected pretreatment, while no change was observed in ULK1.

Since ULK2 was elevated in regenerated cells, we asked whether inhibition of ULK2 would impede cell regeneration. We repeated our in vitro relapse model with the ULK1/2 inhibitor MRT68921 in addition to Ara-C treatment. This combination treatment prevented cell regeneration, and no viable cells were detected at the end of the experiment, which was arbitrarily set at 32 days after the start of the experiment. In comparison, cells treated with Ara-C alone or MRT68921 alone both reached a pretreatment level of viability 16–20 days after the start of the experiment (Figure 6b).

Given that ULK2 appears to be important in maintaining SORE6^+^ cells, but not SORE6^−^ cells, we asked whether cells regenerated from MRT68921 treatment alone were enriched with SORE6^−^ cells. As shown in Figure 6c, cells that regenerated from MRT68921 treatment were composed entirely of SORE6^−^ cells. In contrast, cells regenerated from Ara-C treatment were enriched with SORE6^+^ cells. This finding may explain why cell regeneration was inhibited by combining MRT68921, as it targets the SORE6^+^ cell subset, which is the major culprit of relapse in our in vitro model.

### 2.8. Relapsed Bone Marrow Samples Express Higher ULK2 Compared with Initially Diagnosed Specimens

**Figure 7:**
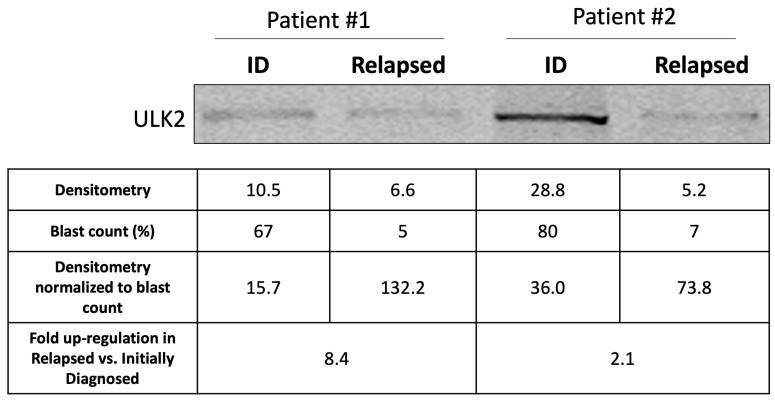
Relapsed AML bone marrow specimens showed higher ULK2 expression compared to initially diagnosed bone marrow specimens. Results from Western blot studies and densitometry analysis of two paired patient samples with both initially diagnosed and relapsed specimens. The densitometry data of the ULK2 bands were normalized to the blast count, and the fold-upregulation was calculated for each patient.

Given that a high expression of ULK2 was found to be important in the ‘in vitro relapse’, we compared the expression of ULK2 in AML patient samples before treatment and at relapse. Using Western blot studies of bone marrow samples from the initially diagnosed and relapsed specimens from the same patient (*n* = 2), we found that ULK2 was higher in the relapsed samples, after the normalization for the blast count was performed (Figure 7).

## 3. Discussion

There is accumulating evidence supporting the role of autophagy as a promoter of chemoresistance in cancers [22]. Regarding FLT3-ITD AML, one published study has shown that inhibition of autophagy using shRNA targeting ATG12 or ATG5 can significantly reduce cell proliferation in FLT3-ITD cell lines or their tumorigenicity in a mouse model [23]. In another study, it was found that inhibiting autophagy through ULK1/2 pharmacological inhibitors (MRT68921 and SBI-0206965) can effectively induce apoptosis in FLT3-ITD AML cell lines [24]. Furthermore, inhibition of autophagy using shATG5 sensitized FLT3-ITD cells to the FLT3 inhibitor AC220 [25]. Interestingly, in all of these studies, the antileukemic effects after autophagy inhibition were only observed in FLT3-ITD, but not FLT3 wild-type, AML cell lines and patient samples. For this reason, in our study, we chose to use two FLT3-ITD cell lines. The mechanisms underlying these observations have not been comprehensively studied. None of these studies examined the role of autophagy-mediated chemoresistance in CSL and bulk cells separately.

Cancer stem cells (CSCs) and CSL cells are believed to be the major contributors to cancer relapse [26]. Thus, it is highly important and relevant to understand how cancer stemness is regulated. In recent years, evidence that autophagy is one of the important mechanisms in promoting cancer stemness has been accumulating. In support of this concept, several published studies have shown that inhibition of autophagy in CSCs/CSL cells can effectively attenuate their stemness, as mentioned in the Introduction [18]. Attenuation of CSL features resulting from autophagy blockade was also reported in pancreatic cancer and osteosarcoma models [27,28]. The results from our current study are also consistent with the concept that autophagy is a key contributor to cancer stemness. Thus, inhibition of autophagy using chloroquine or the ULK1/2 inhibitor, MRT68921, significantly decreased chemoresistance to Ara-C in SORE6^+^ cells. The lack of a significant response in the SORE6^−^ cell population to chloroquine and MRT68921 increases the specificity of our findings.

Studies of cancer relapse have been highly challenging due to the relative paucity of in vitro models and the difficulty in obtaining a sufficient number of cancer cells from patients during their remission. However, evidence has been emerging from studies of AML that support the role of cancer stemness in relapse. For instance, one study showed a correlation between the CSC gene signature score of AML bone marrow specimens and relapse rate [29]. Furthermore, several studies comparing initially diagnostic and relapsed bone marrow specimens have provided evidence that cancer stemness increases at relapse. Specifically, one study found that the percentage of CSCs, which were defined using an in vivo limiting dilution assay, increased at relapse by 9- to 90-fold compared to diagnosis [30]. Additionally, a study by Shlush et al. (2017) used whole-genome sequencing of paired diagnosis and relapse blasts to track the origin of clones at relapse [31]. In this study, two major patterns of relapse were identified: one where the dominant relapse clone emerged from the CSC subset, and another where relapse originated from bulk leukemic cells that carried a strong CSC gene signature score. Although two different patterns of relapse emerged, both patterns highlighted the importance of stemness in this process. While these studies provide evidence that cancer stemness contributes to AML relapse, very little is known about the role of autophagy in AML relapse. However, there are a handful of studies on solid tumors providing correlational evidence to support the link between autophagy and cancer relapse. For instance, a high ULK1 protein expression detectable by immunohistochemistry in gastric carcinomas was found to significantly correlate with a high rate of disease relapse [32]. Similarly, in a cohort of breast cancer patients, those with tumors carrying high *ULK1* mRNA expression were found to have a significantly shorter relapse-free survival [33]. Leveraging the SORE6^−^/SORE6^+^ dichotomy, we recently generated an in vitro AML model that mimics the disease clinically. As mentioned in the Introduction, regeneration of AML cells (i.e., in vitro relapse) was identified under certain circumstances after a period of chemotherapy-induced ‘in vitro remission’. Using molecular barcoding, we found that regenerated cells are enriched in SORE6^+^ cells at the expense of SORE6^−^ cells. In the current study, by employing this in vitro model, we provided evidence that pharmacologic inhibition of autophagy using chloroquine or the ULK1/2 inhibitor, MRT68921, can effectively inhibit ‘in vitro relapse’. This finding also correlates well with our observation that the MRT68921 can potently inhibit the expression of Myc, shown to be the key driver of the SORE6 reporter and the associated CSL phenotype. Taken together, it appears that autophagy maintains the stemness in CSCs/CSL cells, which contribute to cancer relapse. Accordingly, inhibition of autophagy, which attenuates cancer stemness, inhibits cancer relapse.

In our model, ULK2 but not ULK1 appears to contribute to the high autophagic flux in SORE6^+^ cells. In the literature, ULK2 has been ‘overshadowed’ by its homologue ULK1, as the former has not been extensively studied. Nonetheless, it is known that the structures of both ULK1 and ULK2 are very similar, with a 98% query cover [21]. Furthermore, both ULK1 and ULK2 contain a N-terminal serine/threonine kinase domain and a C-terminal interacting domain. Both ULK1 and ULK2 proteins also are known to be capable of inducing autophagy by binding to Atg13 and FIP200 [21,34], a process that can be inhibited by mTOR and increased by AMPK [35,36]. Accordingly, ULK1 and ULK2 are generally regarded as being functionally redundant, and evidence to support this redundancy has been shown in several studies. For instance, in mouse embryonic fibroblasts, disruption of autophagy occurred when both ULK1 and ULK2 proteins were suppressed, but not when only either ULK1 or ULK2 was knocked out [37]. Similarly, while ULK1/ULK2 double-knockout mice die within one day after birth, single ULK1 or ULK2 knockout mice had normal survival [38,39,40]. However, results from a few studies suggest that ULK1 and ULK2 may have nonredundant functions. For example, ULK2, but not ULK1, interacts with p62 and WIPI2, and transcriptional regulators [34]. Another study found distinct functions of ULK1 and ULK2 in lipid metabolism, as knockdown of ULK1 inhibited fatty acid oxidation, while knockdown of ULK2 increased it [41]. The results from our current study provide additional evidence that ULK2 carries functions distinct from ULK1. Based on the structural differences in the C-terminal interacting domain between the two ULK proteins, one may speculate that the nonredundant functions are related to the differences in their binding partners. Since ULK2 expression was not increased in SORE6^−^ cells in our study, we would not have observed the importance of ULK2 without using the SORE6^−^/SORE6^+^ dichotomy, highlighting the importance of incorporating the concept of intratumoral heterogeneity into our study model. One of the limitations of our study is that we did not specifically inhibit ULK2, as MRT68921 inhibited both ULK1 and ULK2 expression. Specific inhibition of ULK2 and ULK1 separately would produce more conclusive results that these two proteins act distinctly, and that the promotion of chemoresistance and ‘in vitro relapse’ is attributed to ULK2, rather than ULK1. However, since we found that the increase in expression after Ara-C treatment was exclusive to ULK2, and was not observed with ULK1, we believe this strongly supports that ULK1 and ULK2 are distinct.

In contrast with the ‘general’ autophagy inhibitors such as chloroquine and bafilomycin, MRT68921 was developed in 2015 as a more specific autophagy inhibitor that functions by targeting/suppressing the kinase activity of ULK1/2 proteins [42]. Since MRT68921 was only developed relatively recently, clinical data about its therapeutic efficacy are not available. However, its potential therapeutic effects against cancers have been tested using in vivo animal models. Specifically, mice xenografted with a gastric cancer cell line treated with MRT68921 had significantly lower tumor volume compared to the DMSO treatment group [43]. In view of the efficacy of chloroquine demonstrated in several clinical trials [44], we believe that MRT68921 holds promise as a useful anticancer agent, especially knowing that it can target CSL cells.

To conclude, our findings support that enhanced autophagy contributes to chemoresistance in SORE6^+^ cells, which is not observed in SORE6^−^ cells. This enhanced autophagy may contribute to AML relapse. ULK2 appears to be a key player in enhancing autophagy-mediated chemoresistance in SORE6^+^ cells and may contribute to cancer stemness and relapse. Given our findings, targeting autophagy may be useful in the treatment of AML patients and may hinder relapses.

## 4. Materials and Methods

### 4.1. Generation of SORE6^−^ and SORE6^+^ Cell Clones and Cell Culture

SORE6^−^ and SORE6^+^ cell clones were generated for two FLT3-mutated cell lines, MOLM−13 (CVCL_2119; DSMZ, Braunschweig, Germany) and MV4−11 (CVCL_0064; ATCC, Manassas, VA, USA). Cell lines underwent lentiviral transduction with the SORE6^−^ mCMVo-dsCop-GFP-PURO (SORE6) reporter (National Cancer Institute, NIH, Bethesda, MD, USA) [5]. SORE6 activity, detectable by GFP expression, was assessed using flow cytometry. SORE6^−^ and SORE6^+^ clones were purified using a flow cytometric cell sorter (Sony MA900, Sony Biotechnology, San Jose, CA, USA) based on their GFP expression. All cells transduced with the SORE6 reporter were cultured in Roswell Park Memorial Institute (RPMI) 1640 media (Gibco, Waltham, MA, USA) supplemented with 10% fetal bovine serum (Gibco) and 1% penicillin and streptomycin (Gibco) in the presence of 0.25 µg/mL puromycin.

### 4.2. Antibodies and Drug Treatments

Primary antibodies used in Western blot studies included anti-LC3B antibody (#L7543, 1:1000) from Sigma-Aldrich (Burlington, MA, USA), anti-MYC (Y69, #ab32072, 1:1000) from Abcam (Cambridge, MA, USA), anti-ULK1 (D8H5, #8054, 1:1000) from Cell Signaling Technology (Danvers, MA, USA), anti-ULK2 (#PA5-22173, 1:1000) from ThermoFisher Scientific (Waltham, MA, USA), and anti-β-actin (#sc-47778, 1:2000) from Santa Cruz Biotechnology (Santa Cruz, CA, USA). MRT68921 (#S7949), Ara-C (U-19920A, #S1648), and chloroquine (#S6999) were purchased from Selleckchem (Houston, TX, USA). IRDye 800CW goat anti-rabbit IgG (#926-32213, 1:40,000) and anti-mouse IgG (#926-32212, 1:40,000) were used as the secondary antibody (LI-COR Biosciences, Lincoln, NE, USA).

### 4.3. Western Blot

Cell pellets were lysed with RIPA buffer (MilliporeSigma, Burlington, MA, USA), with protease and phosphatase inhibitors (MilliporeSigma). Proteins were separated on a 10–15% polyacrylamide SDS-PAGE gel, transferred to a nitrocellulose membrane (GE Healthcare, Velizy-Villacoublay, France), and then incubated with primary antibodies. The membrane was then incubated with horseradish peroxidase-conjugated secondary antibodies. Bands were visualized using an Odyssey^®^ Infrared Imaging System (LI-COR, Lincoln, NE, USA).

### 4.4. Autophagy Assay Red Detection Kit

Cells were incubated with autophagy probe dye (BioRad, Hercules, CA, USA) for 30 min at room temperature. The fluorescence from the dye was detected using flow cytometry using a BD LSRFortessa X-20 Cell Analyzer (BD, Franklin Lakes, NJ, USA) as indicated by the manufacturer.

### 4.5. Cell Viability Assay

Cells were plated with a concentration of 250,000 cells/mL of media with 5% fetal bovine serum. Cell viability was assessed using trypan blue exclusion. Viable cells, defined as trypan blue-negative cells, were counted using direct microscopic examination. IC50 was calculated with GraphPad Prism version 8 software (GraphPad Software, San Diego, CA, USA) using a nonlinear regression curve.

### 4.6. RNA Extraction and Quantitative Real-Time Polymerase Chain Reaction

RNA was extracted from cell lines using the RNeasy Plus Mini Kit (Qiagen, Valencia, CA, USA) as indicated by the manufacturer’s protocol. cDNA conversion of RNA was performed with the High-Capacity cDNA Reverse Transcription Kit (Invitrogen, Waltham, MA, USA). Quantitative real-time polymerase chain reaction (qRT-PCR) was carried out using the Power SYBR™ Green Master Mix (ThermoFisher Scientific, Waltham, MA, USA) with the following primers:

ULK1 F-5-CCTCGCCAAGTCTCAGACGC-3 & R-5-CCCCACCGTTGCAGTACTCC-3
ULK2 F-5-CTCCTCAGGTTCTCCAGTGC-3 & R-5-TTGGTGGGAGAAGTTCCAAG-3
GAPDH F-5-GGAGCGAGATCCCTCCAAAAT-3 & R-5-GGCTGTTGTCATACTTCTCATGG-3

The PCR reactions were quantified using the QuantStudio™ 5 (ThermoFisher Scientific). Gene expression was normalized to GAPDH expression.

### 4.7. Autophagy Array

cDNA used for the autophagy array was generated as described above (Section 4.6). The RT2 Profiler™ PCR Human Autophagy Array (PAHS-084Z, Qiagen, Germantown, MD, USA) was employed as indicated by the manufacturer to analyze gene expression in 84 autophagy related genes.

### 4.8. In Vitro AML Relapse Model for Relapses

We previously generated an in vitro model for disease relapses [13]. Cells were plated to a concentration of 150,000 cells/mL and were treated with 500 nM Ara-C for two days, which induced ‘in vitro remission’, defined by the absence of trypan blue-negative cells. After two days of treatment, media without Ara-C was added to the culture. To detect regeneration, 200 µL of the cell culture was removed for trypan blue cell counting every two days, and the cell culture was replenished with 200 µL of fresh culture media. The experiment end was arbitrarily set 32 days after the start of the experiment or once the cell density reached the same level as the original density. If no viable cells were detected by day 32, the cells were considered unable to regenerate.

### 4.9. Patient Samples

Bone marrow aspirates representing the initially diagnostic sample as well as relapse samples from two FLT3-mutated AML patients were retrieved retrospectively from the University of Alberta Hospital. The use of these patient samples was approved by the Health Research Ethics Board of Alberta (HREBA.CC-21-0253_REN1; date of approval 7 July 2022).

### 4.10. Statistical Analysis

Statistical analyses were performed using GraphPad Prism 8 (Graphpad Software Inc., La Jolla, CA, USA). *p*-values were calculated using two-tailed Student’s *t*-test.

## Data Availability

The data presented in this study are available in the article and supplemental material.

## Supplementary Materials

The following supporting information can be downloaded at: https://www.mdpi.com/article/10.3390/ijms25010646/s1.
