# Supplementary material for: ULK2 Is a Key Pro-Autophagy Protein That Contributes to the High Chemoresistance and Disease Relapse in FLT3-Mutated Acute Myeloid Leukemia
Source: Int J Mol Sci. 2024 Jan 4;25(1):646. doi: 10.3390/ijms25010646 (PMC10780038; doi:10.3390/ijms25010646)
Supplement: Supplementary file 1 [file ijms-25-00646-s001.zip › ijms-2740499-supplementary.pdf]

SORE6+/SORE6- No Ara-C		SORE6+/SORE6- With 10 nM Ara-C	
**Gene**	**Fold Up- or Down-Regulation**	**Gene**	**Fold Up- or Down-Regulation**
ESR1	10.90	***ULK2***	13.90
FAS	9.19	*INS*	9.03
PTEN	9.08	*SNCA*	8.37
SNCA	8.55	*IFNG*	7.89
MAPK14	8.47	*APP*	7.61
APP	8.07	*TMEM74*	7.20
IFNG	7.26	*DAPK1*	5.03
CTSS	6.83	*TGM2*	4.37
ATG10	6.79	*IGF1*	3.42
BNIP3	6.76	*ESR1*	3.41
MAP1LC3B	6.73	*ATG9B*	3.15
TMEM74	6.65	*BNIP3*	2.51
AKT1	6.62	*CTSS*	2.44
DAPK1	6.26	*MAP1LC3A*	2.25
ULK2	5.67	*ATG10*	2.15
INS	5.09	*RAB24*	2.14
TGM2	5.04	*PIK3R4*	1.99
CDKN2A	4.60	*BAK1*	1.87
RAB24	4.19	*HTT*	1.76
ATG9B	4.01	*ATG16L2*	1.69
CTSB	3.90	*CTSD*	1.68
MTOR	3.76	*SQSTM1*	1.67
GABARAPL2	3.51	*NFKB1*	1.60
MAP1LC3A	3.28	*HSPA8*	1.56
BAK1	3.26	*EIF4G1*	1.55
PIK3R4	3.10	*GABARAPL1*	1.51
MAPK8	3.02	*DRAM1*	1.48
ATG7	2.89	*AMBRA1*	1.46
HSP90AA1	2.35	*UVRAG*	1.46
BID	2.32	*MAPK14*	1.46
ATG4B	2.25	*CTSB*	1.45
HSPA8	2.09	*GAA*	1.44
IGF1	2.06	*BCL2L1*	1.43
EIF4G1	2.04	*RPS6KB1*	1.42
ATG12	1.99	*TGFB1*	1.42
CASP8	1.98	*NPC1*	1.41
ATG5	1.94	*CASP8*	1.41

EIF2AK3	1.87	*IRGM*	1.41
AMBRA1	1.87	*LAMP1*	1.40
BCL2L1	1.79	*PIK3C3*	1.40
DRAM1	1.70	*ATG4C*	1.37
RPS6KB1	1.65	*PRKAA1*	1.37
GABARAPL1	1.63	*HSP90AA1*	1.36
WIPI1	1.62	*HDAC1*	1.36
PRKAA1	1.51	*BECN1*	1.35
ATG16L1	1.51	*ATG4B*	1.34
ATG16L2	1.50	*ATG7*	1.34
PIK3C3	1.49	*MAP1LC3B*	1.34
CASP3	1.49	*PIK3CG*	1.33
PIK3CG	1.41	*PTEN*	1.33
FADD	1.38	*MTOR*	1.32
IRGM	1.37	*ATG16L1*	1.30
NPC1	1.35	*TP53*	1.29
ATG4D	1.33	*ATG9A*	1.29
HTT	1.30	*CLN3*	1.28
SQSTM1	1.30	*EIF2AK3*	1.26
ATG3	1.28	*FADD*	1.25
NFKB1	1.22	*ATG5*	1.25
ATG4C	1.20	*TNFSF10*	1.24
BECN1	1.17	*HGS*	1.22
DRAM2	1.14	*WIPI1*	1.21
GAA	1.14	*GABARAPL2*	1.20
ATG4A	1.14	*CASP3*	1.20
BAD	1.10	*ATG12*	1.19
CXCR4	1.09	*ATG4A*	1.18
TP53	1.08	*ULK1*	1.16
UVRAG	1.08	*HDAC6*	1.14
HDAC1	1.07	*BID*	1.13
RB1	1.06	*RB1*	1.12
HDAC6	1.01	*MAPK8*	1.07
BAX	-1.02	*BCL2*	1.07
LAMP1	-1.03	*AKT1*	1.06
ULK1	-1.08	*CXCR4*	1.06
CLN3	-1.08	*BAX*	1.05
CTSD	-1.14	*DRAM2*	1.05
ATG9A	-1.15	*ATG3*	1.05
TNFSF10	-1.21	*BAD*	1.02
GABARAP	-1.26	*RGS19*	1.01

CDKN1B	-1.33	*ATG4D*	-1.02
BCL2	-1.35	*CDKN1B*	-1.02
TGFB1	-1.35	*GABARAP*	-1.03
HGS	-1.36	*TNF*	-1.42
RGS19	-1.38	*CDKN2A*	-1.46
TNF	-1.42	*FAS*	-3.32

Table S1. List of differential gene expression for all genes analyzed by the oligonucleotide array. The fold up- and down- regulation is indicated for MOLM-13 SORE6$^+$ compared to SORE6$^-$ cells, with and without Ara-C treatment.
